# Cross-Cultural Adaptation and Validation of the Persian Version of the Harris Hip Score

**DOI:** 10.1016/j.artd.2023.101180

**Published:** 2023-09-09

**Authors:** Peyman Mirghaderi, Amirhossein Ghaseminejad-Raeini, Alireza Azarboo, Reza Mirghaderi, Hadi Ravanbod, S.M. Javad Mortazavi

**Affiliations:** a Surgical Research Society (SRS), Students' Scientific Research Center, Tehran University of Medical Sciences, Tehran, Iran; b Joint Reconstruction Research Center, Tehran University of Medical Sciences, Tehran, Iran; c Department of Orthopedic Surgery, Al-Zahra Hospital, Isfahan University of Medical Sciences, Isfahan, Iran

**Keywords:** Arthroplasty, Harris hip score, Persian, Questionnaire, Reliability, Validity

## Abstract

**Background:**

The Persian language, also known as Farsi, is a pluricentric language spoken in Iran, Afghanistan, and Tajikistan by about 140 million people. This study aims to translate the Harris hip score (HHS) into Persian with cross-cultural adaptation and to evaluate its validity and reliability.

**Methods:**

One hundred fifty-six total hip arthroplasty patients completed the Persian version of the HHS, Western Ontario and McMaster Universities Osteoarthritis Index, Forgotten Joint Score, and visual analog scale (VAS) for pain and satisfaction postoperatively. Using Cronbach’s alpha (α) coefficient, internal consistency was evaluated. Correlations (Spearman's Rho) were used to assess validity. A test-retest reliability assessment of the Persian HHS was conducted (n = 47) using the intraclass correlation coefficient. Content validity was evaluated using the floor and ceiling effects of the HHS.

**Results:**

The final translation of the Persian HHS was approved to be used. The preoperative and postoperative Cronbach's alpha were 0.71 and 0.70, respectively, and showed acceptable internal consistency. The intraclass correlation coefficient was excellent (0.869, *P* < .001). Insignificant ceiling effects (13.5%) and no floor effects (0) were observed. The HHS score was significantly and strongly correlated with Western Ontario and McMaster Universities Osteoarthritis Index (r = 0.696, *P* < .001), VAS pain (r = 0.654, *P* < .001), VAS satisfaction (r = 0.634, *P* < .001), and Forgotten Joint Score (r = 0.648, *P* < .001).

**Conclusions:**

The Persian HHS demonstrated excellent reliability and validity properties. Accordingly, Persian HHS may be a helpful tool for assessing patients undergoing total hip arthroplasty.

## Introduction

There are many methods of evaluating the success of a total hip arthroplasty (THA), such as complications rate, revision rate, and patient-reported outcomes measures (PROMs) [[1], [2], [3]]. Given the importance of evaluating hip function after THA, several PROMs have been proposed, including the Harris hip score (HHS), Hip Disability and Osteoarthritis Outcome Score [4], Oxford hip score [5], Lequesne index of severity for osteoarthritis of the hip [6], and American Academy of Orthopedic Surgeons Hip and Knee Questionnaire [7]. Despite being the oldest scoring system, HHS remains well-known for its widespread use across studies [8]. Published in 1969 [9], HHS was designed to assess hip surgery outcomes and a broad range of hip disabilities in an adult population.

A translation and validation of the HHS have been performed in Italian [10], Turkish [11], Arabic [12,13], Portuguese [14], Spanish [15], Greek [16], and Slovenian [17] languages. The Persian language, also known as Farsi, is a pluricentric language spoken primarily in Iran, Afghanistan, and Tajikistan. A total of 140 million people live in countries whose official language is Persian. Despite these individuals' high prevalence of hip disorders, no Persian version of HHS has been reported. Thus, with the absence and growing demand for a Persian version of HHS, our study aims to translate HHS into Persian with cross-cultural adaptation so that Persian-speaking patients also benefit from the merits of this scoring system, as it is the most widely used globally.

## Material and methods

### Study design

A cross-sectional study was carried out in our tertiary referral center (Imam Khomeini hospital, Tehran, Iran) from 2019 to 2020. Inclusion criteria entailed that all adults aged 18 years and older who spoke, read, and wrote Persian underwent primary THA and had more than 1 year of follow-up. Exclusion criteria entailed patients having undergone revision surgery and lacking the mental ability to answer the questionnaire.

### Translation and cross-cultural adaptation

**Figure 1:**
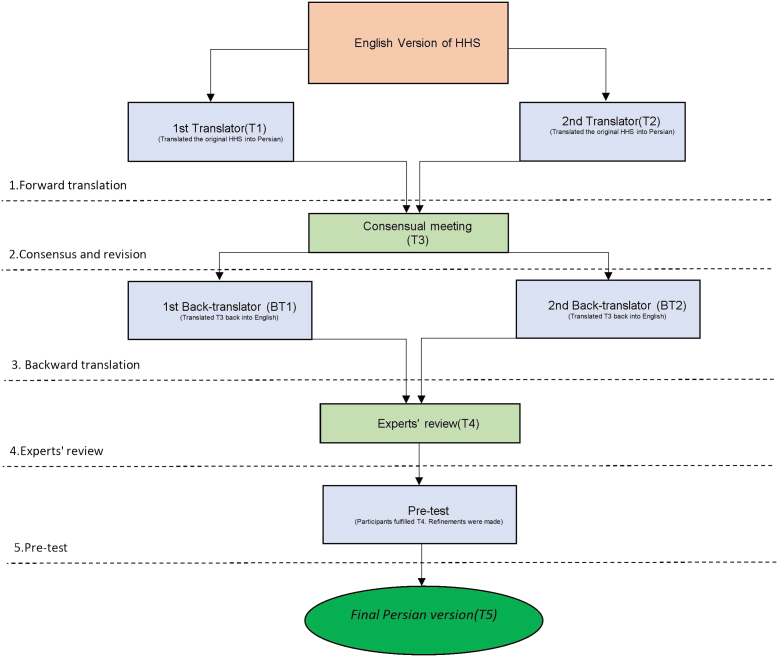
Flow diagram of the study process and enrollment of participants.

Guillemin *et al.* [18] provided a guideline to translate and cross-culturally adapt the scoring systems. We adopted the guidelines above by carefully completing each step. Our study comprises 2 stages: translation of the questionnaire to Persian and back-translation to English, and data collection from patients for reliability and cross-cultural adaptivity. These steps included forward translation, consensus and revision, backward translation, expert review, and pretest [Fig. 1].

### Forward translation

Two well-versed bilingual (fluent in English and Persian) translators were deployed to translate and culturally adapt the original HHS; both qualified to account for the cultural discrepancy between communities [19]. The first translator (T1) was well-versed in medical terminology, familiar with orthopaedics, and knowledgeable about the concept of the study. On the other hand, the second translator (T2) did not have a medical background and had no prior experience in this field.

### Consensus and revision

The consensus was reached as a meeting was held with the experts’ committee comprised of 3 hip surgeons and 2 expert reviewers to revise the primitive versions (T1 and T2) and draw comparisons between the 2. As a result, a consensual Persian version of the HHS was presented (T3).

### Backward translation

The back-translation from Persian to English was performed by another 2 well-qualified bilingual and bicultural translators (BT1 and BT2). Translators were kept blinded as they had utterly no information about the existence or aim of the study, hence a strict blind method. Therefore, the 2 back-translated versions (B1 and B2) entered the next stage.

### Experts’ review

A second meeting was held with the experts' committee, in which they had all translated and back-translated versions (T1, T2, T3, B1, and B2) under rigorous scrutiny. Suggestions were noted, and further alterations were made to refine the questionnaire. Finally, given the unanimous decision among experts, a final Persian version (T4) of HHS was yielded.

### Pretest

In the final stage, we had 20 patients complete the Persian version of HHS (T4) while we asked them about the meaning and clarity of each question. Orthopaedic residents completed objective parts of HHS. We recorded any suggestions they had regarding the structural or functional format of the questionnaire. T4 was adjusted and refined according to participants’ responses. The final version (T5) was collected and ready to be used.

### Patients and scores

A total of 156 THA patients were included. Patients were asked to complete the Persian version of the HHS, Western Ontario and McMaster Universities Osteoarthritis Index (WOMAC) [20], Forgotten Joint Score (FJS) [3], visual analog scale (VAS) [21] for pain and satisfaction postoperatively.

HHS is a scale with a maximum of 100 points administered by a physician or physiotherapist. There are 4 domains and 10 items in the HHS: pain (1 item, 0-44 points), function (7 items, 0-47 points), absence of deformity (1 item, 4 points), and range of motion (ROM) (2 items, 5 points) [22]. The pain domain is used to assess the severity of pain and its impact on daily activities while questioning whether pain medication is required. Second, the function domain is subcategorized into daily activities, climbing stairs, using public transportation, limping, support needed, and walking distance. Third, hip fixed flexion, adduction, internal rotation, and limb length discrepancy are all checked in the deformity domain. Fourth, the ROM domain examines hip ROM. A score lower than 70 is interpreted as a poor result, with 70-80 being fair, 80-90 being good, and 90-100 being excellent [10,23].

We selected 3 valid scores to assess their correlation with the HHS: WOMAC [20], FJS [24], and VAS pain and satisfaction [25]. The HHS has a marked correlation with the WOMAC scale [17], VAS [17], and The Persian FJS (*r* = 0.8, *P* = .001) [26].

### Reliability

Using Cronbach’s alpha (α) coefficient, internal consistency was evaluated [27]. A test's internal consistency indicates how closely it measures the same concept or construct. An α coefficient of 0.70-0.95 was considered appropriate. Test-retest reliability is defined as 2 administrations of the questionnaire during 2 weeks when no change in the target concept has occurred and represents consistency. The intraclass correlation coefficient (ICC) was utilized to assess the test-retest reliability of the Persian HHS. A correlation coefficient of >0.40 was considered satisfactory, and one of >0.80 was considered excellent [28]. Also, the Bland-Altman plot was applied to visualize the agreement between the test and retest responses to the HHS.

### Validity

Construct validity is normally defined as having at least 75% of patients with a THA score of 70 or more at HHS. Of the 3 posited hypotheses by Terwee [29], we employed the second hypothesis: The correlation between correspondent domains in WOMAC/VAS and HHS should be more significant than that of different domains (ie, HHS pain vs VAS pain greater than HHS pain vs VAS patient satisfaction) [10].

Criterion validity keeps the correlation strength through Spearman’s Rho between a measure and a “gold standard” in check. We adopted WOMAC, FJS, and VAS as gold standards. We considered Spearman’s Rho at values >0.55 (confidence interval of 99%) as the acceptable cutoff [29,30].

Content validity was evaluated using the floor and ceiling effects of the HHS. To evaluate them, the proportion of patients scoring a minimal score of 0 or a maximal score of 100 was calculated relative to the number of patients. A floor and ceiling effect was considered relevant if at least 30% of the patients had this effect. It is considered good content validity if less than 15% of results achieve minimum or maximum values [28].

### Ethical consideration

This study was approved by the institutional review board of our university of medical sciences (IRB Approval ID: IR.TUMS.IKHC.REC.1401.417). Verbal approval was obtained from each participant after they were informed of the purpose of the study. We have obtained permission to conduct this research study and translation without requiring explicit permission from the authors of the original questionnaire through email communication.

### Statistical analysis

Statistical Package for Social Studies (SPSS 23; IBM Corp, New York, NY) was used to analyze our data. Mean ± standard deviation expressed continuous variables, and frequency demonstrated categorical variables. Spearman’s Rho correlation coefficient was used to assess the correlation between HHS and WOMAC, FJS, and VAS. Cronbach’s alpha was used to determine the reliability and internal consistency of the items in the HHS questionnaire [31].

## Results

### Cross-cultural adaptation

The 2 mentioned translators and back-translators did the translation process accurately and wrote the primary version of the questionnaire. Consensus refinements were made and resolved by the professional expert team. The first draft was pretested among 20 patients, and further comments were recorded. Alongside that, the experts meeting voted anonymously on the clarity of each question regarding the first version of HHS. Eventually, no major corrections needed to be addressed, and the final translation of the Persian HHS was approved to be used in the following steps.

### Patients’ characteristics

The mean age and follow-up of the population were 39.7 ± 13.1 years and 2.1 years, respectively, (range: 1-3 years). Most of them were female (55.8%) and currently married (64.1%). Mean body mass index and Charlson comorbidity index were 24.8 and 0.43, respectively (Table 1). The most common diagnosis among the patients was developmental dysplasia of hip (29.5%), followed by fracture, avascular necrosis, and primary osteoarthritis. The mean postoperative HHS, WOMAC, VAS (pain), VAS (patient satisfaction), and FJS were 86.6, 80.9, 1.9, 9.1, and 71.9.

**Table 1:** Characteristics of the study population (mean ± SD or n [%]).

Variable	
Age, y	39.7 ± 13.1
Sex	
Male	69 (44.2%)
Female	87 (55.8%)
Marital status	
Single	50 (32.1%)
Married	100 (64.1%)
Widowed	3 (1.9%)
Divorced	3 (1.9%)
Charlson comorbidity index	0.43 ± 0.74
BMI, kg/m^2^	24.8 ± 4.4
Diagnosis	
AVN	32 (20.5%)
DDH	46 (29.5%)
Fracture	34 (21.8%)
OA	25 (16.0%)
RA	8 (5.1%)
Other	11 (7.1%)
Postoperative HHS	86.6 ± 16.4
Postoperative WOMAC	80.9 ± 17.5
Postoperative VAS (pain)	1.9 ± 2.4
Postoperative VAS (satisfaction)	9.1 ± 1.8
Postoperative FJS	71.9 ± 34.9

SD, standard deviation; BMI, body mass index; AVN, avascular necrosis; DDH, developmental dysplasia of hip; OA, osteoarthritis; RA, rheumatoid arthritis; HHS, Harris hip score; WOMAC, Western Ontario and McMaster Universities Arthritis Index; VAS, visual analog scale; FJS, Forgotten Joint Score.

### Reliability

Concerning internal consistency, preoperative and postoperative Cronbach’s alpha were calculated as 0.71 and 0.70, respectively. This was above the acceptable limit for the reliability of an outcome measurement tool (more than 0.7), so the translated version possessed a good level of internal consistency. After alternating removal of each item of the scale, Cronbach’s alpha was determined; the results are shown in [Table 2].

**Table 2:** Item-total statistics for each item of the questionnaire.

Item	Scale mean if item deleted	Scale variance if item deleted	Corrected item-total correlation	Cronbach's alpha if item deleted
Pain				
Pre	47.63	91.072	0.696	0.761
Post	26.36	126.08	0.567	0.717
Stairs				
Pre	83.40	244.113	0.600	0.685
Post	43.16	320.91	0.747	0.617
Limping				
Pre	77.90	208.802	0.673	0.641
Post	40.84	286.79	0.590	0.585
Shoe-socks				
Pre	83.18	248.664	0.533	0.692
Post	42.64	329.65	0.554	0.630
Walking				
Pre	77.49	207.219	0.595	0.648
Post	39.71	274.45	0.693	0.564
Sitting				
Pre	81.90	250.707	0.527	0.695
Post	41.315	324.274	0.534	0.625
Transportation				
Pre	85.73	262.095	0.487	0.710
Post	44.102	346.036	0.431	0.650
Walking aid				
Pre	76.71	217.564	0.554	0.660
Post	39.704	264.846	0.537	0.576
Deformity				
Pre	83.42	255.278	0.187	0.710
Post	42.565	368.771	−0.239	0.691
Range of motion				
Pre	82.11	250.278	0.665	0.693
Post	41.018	339.233	0.324	0.644

**Figure 2:**
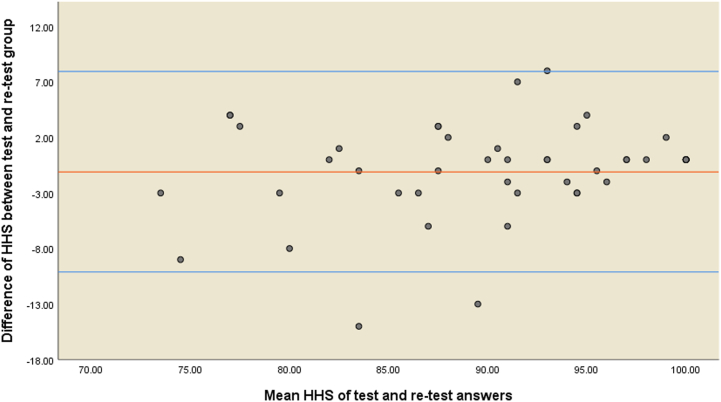
Bland-Altman plot of the Persian Harris hip score.

Forty-seven out of 156 individuals were in aid of retesting the translated HHS to evaluate the test-retest reliability. The ICC was determined to be 0.869 (*P* < .001) between the total HHS scores of the test and retest phases. Regarding the subscales of HHS, the most and least significant correlation was for the deformity (ICC = 0.946, *P* < .001) and sitting on a chair (ICC = 0.499, *P* < .001). Further details are displayed in [Table 3]. All items’ ICC was in the satisfactory and excellent range (r ≥ 0.81). Also, the Bland-Altman plot indicated that nearly all differences between the test and retest total HHS were within the level of agreement (mean difference ±2 standard deviations) [Fig. 2]. Therefore, the Persian version of the questionnaire remained reliable.

**Table 3:** Spearmen correlation coefficients between the test (t) and retest (rt) of the Harris hip score (HHS) to assess the test-retest reliability.

HHS subscales	Pain (rt)	Climbing stairs (rt)	Limp (rt)	Putting on socks or shoes (rt)	Distance walked (rt)	Sitting (rt)	Use of public transportation (rt)	Use of walking supports (rt)	Deformity (rt)	Range of motion (rt)	Total score (rt)
Pain (t)											
*r*	0.674	-	-	-	-	-	-	-	-	-	-
*P*-value	<.001										
Climbing stairs (t)											
*r*	-	0.729	-	-	-	-	-	-	-	-	-
*P*-value		<.001									
Limp (t)											
*r*	-	-	0.888	-	-	-	-	-	-	-	-
*P*-value			<.001								
Putting on socks or shoes (t)											
*r*	-	-	-	0.765	-	-	-	-	-	-	-
*P*-value				<.001							
Distance walked (t)											
*r*	-	-	-	-	0.840	-	-	-	-	-	-
*P*-value					<.001						
Sitting (t)											
*r*	-	-	-	-	-	0.499	-	-	-	-	-
*P*-value						<.001					
Use of public transportation (t)											
*r*	-	-	-	-	-	-	0.569	-	-	-	-
*P*-value							<.001				
Use of walking supports (t)											
*r*	-	-	-	-	-	-	-	0.905	-	-	-
*P*-value								<.001			
Deformity (t)											
*r*	-	-	-	-	-	-	-	-	0.946	-	-
*P*-value									<.001		
Range of motion (t)											
*r*	-	-	-	-	-	-	-	-	-	0.829	-
*P*-value										<.001	
Total score (t)											
*r*	-	-	-	-	-	-	-	-	-	-	0.869
*P*-value											<.001

### Validity

Content validity was evaluated through the floor and ceiling effects. No patients had a 0 score (floor effect). However, 21 out of 156 participants (13.5%) got a full score of 100 (ceiling effect). Researchers had endorsed that the effect size below 15% is fair enough to prove the content validity of a questionnaire. Therefore, the translated version of HHS’s content validity was satisfactory.

Regarding the construct validity, approximately 91% (142/156) of the THA patients scored 70 or more in their follow-up visit. Besides, comparing the correlation coefficients of WOMAC, VAS, and HHS domains indicated that there remained a stronger association between the 2 quite similar parts rather than irrelevant ones. HHS pain score was more significantly correlated to the WOMAC pain domain (r = 0.599) or VAS pain (r = 0.727) than the WOMAC physical function (r = 552) or stiffness (r = 0.351) [Table 4]. Due to these 2 reasons, the Persian version of HHS was considered a valid outcome measure concerning the construct validity.

**Table 4:** Spearmen correlation coefficients between different domains of the Harris hip score (HHS) and Western Ontario and McMaster Universities Arthritis Index (WOMAC), visual analog scale (pain and patient satisfaction), and Forgotten Joint Score (FJS).

HHS subscales	Pain	Climbing stairs	Limp	Putting on socks/shoes	Distance walked	Sitting	Use of public transportation	Use of walking supports	Deformity	Range of motion	Total score
WOMAC (pain)											
*r*	0.599	0.504	0.367	0.322	0.485	0.295	0.384	0.351	0.179	0.374	0.603
*P*-value	<.001	<.001	<.001	<.001	>.001	<.001	<.001	<.001	.026	<.001	<.001
WOMAC (stiffness)											
*r*	0.351	0.371	0.346	0.433	0.243	0.221	0.275	0.270	0.331	0.365	0.411
*P*-value	<.001	<.001	<.001	<.001	.002	.006	.001	.001	<.001	<.001	<.001
WOMAC (physical function)											
*r*	0.552	0.663	0.334	0.497	0.619	0.369	0.384	0.347	0.220	0.492	0.688
*P*-value	<.001	<.001	<.001	<.001	<.001	<.001	<.001	<.001	.006	<.001	<.001
WOMAC (total)											
*r*	0.568	0.635	0.347	0.480	0.616	0.379	0.383	0.356	0.245	0.488	0.696
*P*-value	<.001	<.001	<.001	<.001	<.001	<.001	<.001	<.001	.002	<.001	<.001
VAS (pain)^a^											
*r*	0.727	0.417	0.395	0.307	0.434	0.231	0.314	0.232	0.105	0.296	0.654
*P*-value	<.001	<.001	<.001	<.001	<.001	<.001	<.001	.004	.193	<.001	<.001
VAS (patient satisfaction)											
*r*	0.519	0.499	0387	0.373	0.584	0.319	0.308	0.336	0.214	0.544	0.634
*P*-value	<.001	<.001	<.001	<.001	<.001	<.001	<.001	<.001	.008	<.001	<.001
FJS											
*r*	0.574	0.422	0.455	0.384	0.483	0.355	0.394	0.269	0.261	0.458	0.648
*P*-value	<.001	<.001	<.001	<.001	<.001	<.001	<.001	.001	.001	<.001	<.001

a The absolute value of the correlation was displayed.

Criterion validity was assessed by comparing the HHS and its relevant gold standards. In the present study, it was revealed that the total HHS score was meaningfully associated with WOMAC total score (r = 0.696, *P* < .001), VAS pain (r = 0.654, *P* < .001), VAS satisfaction (r = 0.634, *P* < .001), and FJS (r = 0.648, *P* < .001). This confirmed the acceptable validity of the Persian HHS.

## Discussion

The final version of the Persian HHS revealed no difficulties with comprehension or the cultural adaptation. Additionally, the Persian version of the HHS was found to be reliable and valid when used to evaluate PROMs of Persian-speaking individuals who had undergone THA surgery. The results are similar to those reported for Italian [10], Turkish [11], Arabic [12,13], Portuguese [14], Spanish [15], Greek [16], and Slovenian [17] adaptation studies [Table 5].

**Table 5:** Test-retest reliability, internal consistency, and validity of the Harris Hip Score (HHS) in different languages.

Study and reference	Language	Population	Test-retest reliability (ICC)	Cronbach’s alpha	Validation scores	Correlation (Pearson’s r)
Dettoni 2015 [10]	Italian	103	0.975	0.816	WOMAC	−0.7568
					SF-12	0.563
Alshaygy 2022 [13]	Arabic	100	0.70	0.528	SF-36	0.71
				0.742 (after standardization)		
Al-Qahtani 2021 [12]	Arabic (modified HHS)	183	0.936	0.792	HHS subscales	0.820-0.971
Stasi 2020 [16]	Greek	90	0.881	0.614	LEFS	0.801
					WOMAC	−0.783
					TUG	−0.547
					9S-A/D	−0.575
Josipović 2020 [17]	Slovenian	42	0.983	0.94	WOMAC	−0.877
					VAS	−0.717
					SF-36	0.442-616
Çelik 2014 [11]	Turkish	80	0.91	0.70	OHS	0.75
					WOMAC	0.64
					VAS	0.10-0.72
					SF-36	0.60
Taranchenko 2022 [15]	Spanish	100	-	-	-	-
This study 2023	Persian	156	0.869	0.71	WOMAC	0.696
					VAS pain	0.654
					VAS satisfaction	0.634
					FJS	0.648

LEFS, lower extremity functional scale; WOMAC, Western Ontario and McMaster Universities Arthritis Index; SF-12, 12-Item Short Form Survey; SF-36, 36-Item Short Form Survey; TUG, Timed Up and Go; 9S-A/D, 9-stairs-ascend/descend (9S-A/D); OHS, Oxford hip score; FJS, Forgotten Joint Score.

### Reliability

Cronbach's alpha and test-retest reliability were used to evaluate the reliability of the Persian HHS. Cronbach's alpha indicated that the internal consistency of the preoperative and postoperative HHS was 0.71 and 0.70, respectively, which is above the threshold generally considered acceptable for research purposes (>0.70). The 10 items of Persian HHS are moderately interdependent and homogenous in terms of the constructs they measure. The Cronbach's alpha values for other languages range from 0.528 for Arabic to 0.94 for Slovenian and are consistent with our findings [13,17]. When Cronbach's alpha is calculated if an item is deleted, the Limping score is found to be the most significant item. This may be because our sample consisted of patients with end-stage hip disorders who underwent THA. They are characterized by pain and limited active and passive ROM, which affect hip mobility and contribute to limping and abnormal walking patterns [3]. Also in the Greek version, the Gait-Limp subscale was ranked as the most important item [16].

The test-retest ICC value was 0.869, which is acceptable reliability, while the results for the other languages ranged from 0.7 for Arabic to 0.983 for Slovenian. Kemp *et al.* [32] reported a similar ICC value (=0.91) for English HHS, which is in agreement with ours. According to studies, a PROM can be considered suitable for use in groups (research) if it has an ICC above 0.8 and for use in patients (clinical) if it has an ICC above 0.9 [31,33]. Also, Persian HHS total scores did not exhibit a ceiling or floor effect.

A variety of intervals have been used to study the reliability of test-retest reliability for health-related Quality of Life instruments, ranging from 10 minutes to 1 month [[34], [35], [36]]. Various time intervals have been reported in the literature for estimating test-retest reliability for the HHS, ranging from 7 to 14 days [[34], [35], [36]]. When the disease is measured and is expected to change rapidly, it is necessary to administer a PROM less than 7 days apart [35,36]. The participants in our study did not receive any additional interventions that would have resulted in rapid changes in their condition. All of them were >1-year post-THA. Thus, the authors have chosen time interval of 2 weeks based on similar studies [15,16,35]. A study conducted by Marx *et al.* [35] did not demonstrate a significant difference between measuring test-retest reliability with a 2-day interval as compared to a 2-week interval for athletic patients with disorders. Both intervals are considered short enough to prevent any change in the patient's disease and long enough to prevent the patient from recalling the answers provided in the first place.

### Validity

The constructive validity of the Persian HHS was determined by the strong correlation with WOMAC (r = 0.696), VAS pain (r = 0.654), VAS satisfaction (r = 0.634), and FJS (r = 0.648) (All *P* < .001). In our study, the correlation between WOMAC stiffness score and HHS was the weakest (r = 0.411). This moderate association is because this subscale examines the stiffness a patient experiences after first waking up in the morning and later in the day after resting or sitting. However, HHS assesses function primarily during the activities described.

Stasi *et al.* [16] investigated the Greek modified and similarly found high correlations of HHS with other PROMs, whereas correlations with objective physical performance measures (PPMs) were moderate. As PROMs measure patient perception while PPMs, including the Timed Up and Go test and the 9-stairs ascend/descend test, measure patient ability, this moderate correlation between PROMs and PPMs is not surprising [37]. For a comprehensive understanding of functionality, both assessment methods are needed [37].

Also according to the hypothesis of Terwee *et al.* [29], the correlation coefficients of WOMAC, VAS, and HHS domains indicated that there remained a stronger association between the 2 quite similar parts rather than irrelevant ones (HHS pain, WOMAC pain, and VAS pain). For the parameters we evaluated, it is justified to use the WOMAC since it has been validated and translated worldwide and has shown excellent correlation with the HHS [38].

### Limitations

There were some serious limitations to this study. We did not use generic health status scales like 36-Item Short Form Survey to test the constructive validity of HHS; instead, we used joint-specific scores. Moreover, we only assessed Persian HHS among patients undergoing THA surgery, whereas patients with other conditions, such as hip arthroscopy, were not assessed. Also, we did not examine the content validity or face validity of the Persian HHS. Finally, due to the readministration of the HHS by 2 medical professionals, there may be bias related to the administrator.

## Conclusions

Translating and culturally adapting the HHS into Persian was completed according to existing guidelines. We investigated the validity and reliability of the Persian version of the HHS and concluded that it is valid and reliable. Persian HHS could be used in clinical practice and research to evaluate THA patients. Further research is required to confirm our findings and explore the questionnaire's reliability characteristics across different patient groups as well as its validity characteristics against other PROMs.

## Conflicts of interest

The authors declare there are no conflicts of interest.

For full disclosure statements refer to https://doi.org/10.1016/j.artd.2023.101180.

## Ethical approval

The study was reviewed and approved by the Institutional Review Board of Tehran University of Medical Sciences.

## Consent to participate

An informed consent signed by all the participants of study.

## Consent to publish

Patient consent was obtained regarding publication of data and photographs.

## Author contributions

P.M. and S.M.J.M. contributed to the study conception and design, wrote the draft, and edited the manuscript. A.G.-R. and A.A. contributed to the study design, analyzed the data, data collection, and wrote the first draft of the manuscript. R.M. and H.R. contributed to the study design, revised the manuscript, data collection, and drew figures. All authors commented on previous versions of the manuscript and revised it. All authors read and approved the final manuscript.

## Availability of data and material

The data that support the findings of this study are available from the corresponding author, SM Javad Mortazavi, upon reasonable request.

## Appendix A Supplementary data

Conflict of Interest Statement for Azarboo

Conflict of Interest Statement for Ghaseminejad-Raeini

Conflict of Interest Statement for Ravanbod

Conflict of Interest Statement for Mirghaderi

Conflict of Interest Statement for Reza Mirghaderi

Conflict of Interest Statement for Javad Mortazavi
